# Gastrointestinal Dysbiosis

**DOI:** 10.1093/emph/eou029

**Published:** 2014-11-20

**Authors:** Daniel T. Blumstein, Karen Levy, Emeran Mayer, John Harte

**Affiliations:** ^1^Department of Ecology and Evolutionary Biology, University of California, Los Angeles, CA, USA; ^2^Rollins School of Public Health, Emory University, Atlanta, GA, USA; ^3^Division of Digestive Diseases, University of California, Los Angeles, CA, USA; ^4^Energy and Resources Group, University of California, Berkeley, CA, USA

## Gastrointestinal dysbiosis

Gastrointestinal dysbiosis results from perturbations of the intestinal microbiota from dietary or environmental changes, certain antibiotic therapies, psychosocial stress, exposure to pathogenic organisms or by altered/dysregulated immune responses. Once established during the first few years of life, the intestinal microbial community is remarkably stable. In the majority of perturbations, changes of microbiota composition (both in diversity and abundance) are transient, causing temporary symptoms. However, in the rare cases in which dysbiosis is long-lasting or permanent, chronic symptoms may develop. Thus, dysbiosis can manifest as temporary or chronic clinical symptoms, or be asymptomatic but increase vulnerability for various diseases, including intestinal infections, as well as metabolic and brain diseases.

## Evolutionary perspectives

Dysbiosis can be viewed through the lens of ecology and evolution [1], especially the subfields of community ecology, disturbance ecology and restoration ecology that examine species distributions across landscapes. A stable community is where resident species resist change; in a healthy gut, resident microorganisms generally prevent invasion of pathogenic species [2].

**Figure 1.**
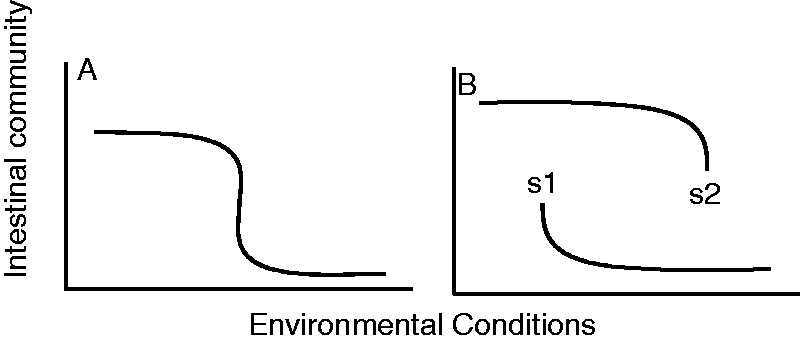
(**A**) Rapid, but continuous change in intestinal community composition under changing environmental conditions. (**B**) Alternative stable states (s1, s2) characterized by discretely different microbial communities can exist under similar environmental conditions

Gut dysbiosis can, however, also be considered a stable state because certain dominant species may change the gut environment or out-compete other species (Fig. 1) [3]. A shift from one stable community (whether healthy or unhealthy) to another can be triggered by the introduction of a novel species, an environmental change or the loss of key species [4]. The presence of a single pathogenic species (e.g. *Escherichia coli*), however, may not signify dysbiosis. Indeed, if the elimination or replacement of that species is sufficient to restore gut health, the existing community is unlikely to have been in a stable state. These distinctions are important for treatment purposes.

## Future implications

Healthy versus disrupted ecological systems have different signatures for species abundance distributions [5]. Thus, the number and types of gut microbiota may have diagnostic potential in determining relevant ‘ecological’ treatments.

Pre-biotic and pro-biotic therapies have been proposed to change gut communities [1], but it might take more than the addition of a few ‘desirable’ bacteria to trigger a recovery. Rather, mass elimination of one stable community may be required before trying to re-introduce an alternative stable, healthy community.

Fecal transplants have been effective against *Clostridium difficile* infection. Combining this therapy with a drastic reduction in the size of the dysbiotic community, coupled with manipulations of environmental factors, such as pH, may result in more rapid colonization of a healthy microbial community.
